# Radicular Cyst: The Sequelae of Untreated Caries

**DOI:** 10.7759/cureus.60269

**Published:** 2024-05-14

**Authors:** Rohan R Khetan, Amit Reche, Arshjot S Basra, Srushti S Awghad

**Affiliations:** 1 Department of Oral Medicine and Radiology, Sharad Pawar Dental College and Hospital, Datta Meghe Institute of Higher Education & Research, Wardha, IND; 2 Department of Public Health Dentistry, Sharad Pawar Dental College and Hospital, Datta Meghe Institute of Higher Education & Research, Wardha, IND

**Keywords:** radiolucency, cortical plates, swelling, periapical cyst, radicular cyst

## Abstract

A radicular cyst is characterized as an odontogenic cyst of inflammatory origin that develops from Malassez epithelial rests in the periodontal ligament as the consequence of dental pulp inflammation. The cyst commenced in the carious tooth and spread to the periodontal and periapical regions. The majority of these lesions appear as precise radiolucencies and encompass their entire apex. The cystic lesion, which is also called a root-end cyst or periapical cyst, is sometimes referred to as a true cyst because it is lined by fluid epithelium. There are several treatment options to address radicular cysts, including surgical and nonsurgical methods. In this case study, we described the clinical observation of the cyst. The cyst typically manifests in later life due to its prolonged etiology. The maxillary anterior region is the most frequently utilized site.

## Introduction

Radicular cysts account for 52% to 68% of cystic lesions. The World Health Organization categorizes jawbone cysts into three groups: inflammatory, neoplastic, and developmental. The word “cyst” originates from the Greek word “kystis,” which means bladder or sac. Histological examination of biopsy specimens can provide a conclusive diagnosis of radicular cysts. Cone-beam computed tomography also helps in the identification of radicular cysts radiographically by assessing the size of the radiolucency and the destruction of the cortical plate. It also shows the involvement of the floor of the nasal cavity by enlarging the radicular cyst. Radicular cysts are thought to be treatable with traditional root canal therapy because they are brought on by root canal infections. Inflammatory odontogenic cysts encompass radicular or lateral periodontal cysts. Radicular cysts are inflammatory lesions of the jaws that are usually located at the apex of the infected tooth [1]. They are believed to have originated from an epithelial rest of Malassez in the periodontal ligaments as a result of inflammation [2,3]. They account for between 52% and 68% of all human cystic lesions and are the most widespread kind of jaw cysts [4]. Radicular cysts are rarely noticed until they are diagnosed by a habit of radiographic examination; however, in certain situations, chronic or long-standing lesions cause the cyst to unexpectedly get worse, causing swelling, pain, and pus discharge [5].

These cysts can develop in the periapical region of any tooth at any age, although they are hardly ever connected to the primary teeth [6]. Compared to the mandible, it occurs more frequently in the maxillary anterior region. The cyst may result in mild root resorption or adjacent tooth displacement [7]. For smaller lesions, customary nonsurgical root canal therapy is an option for treating radicular cysts; for larger cysts, surgical procedures such as enucleation, marsupialization, or decompression are readily accessible [8]. The successful surgical treatment of a large infected radicular cyst is examined in this case study. Radicular cysts are frequently linked to carious teeth or teeth with a history of trauma [9]. This document presents the case of a large maxillary radicular cyst that exhibits a few distinctive features.

Usually radiolucent, radicular cysts have well-defined, expansile, corticated borders that give the impression that they are hydraulic in nature. However, internal calcifications have been documented in a few cases in the literature, which can be confusing and result in a different subset of differential diagnoses and treatment modalities [10]. This case shows how crucial a clinical examination is to get a more accurate differential diagnosis and an appropriate treatment plan, in addition to radiographic features [11]. The radicular cyst in this case has an uncommon radiographic appearance. We describe a case of internal calcifications in a radicular cyst that poses diagnostic difficulties.

## Case presentation

**Figure 1:**
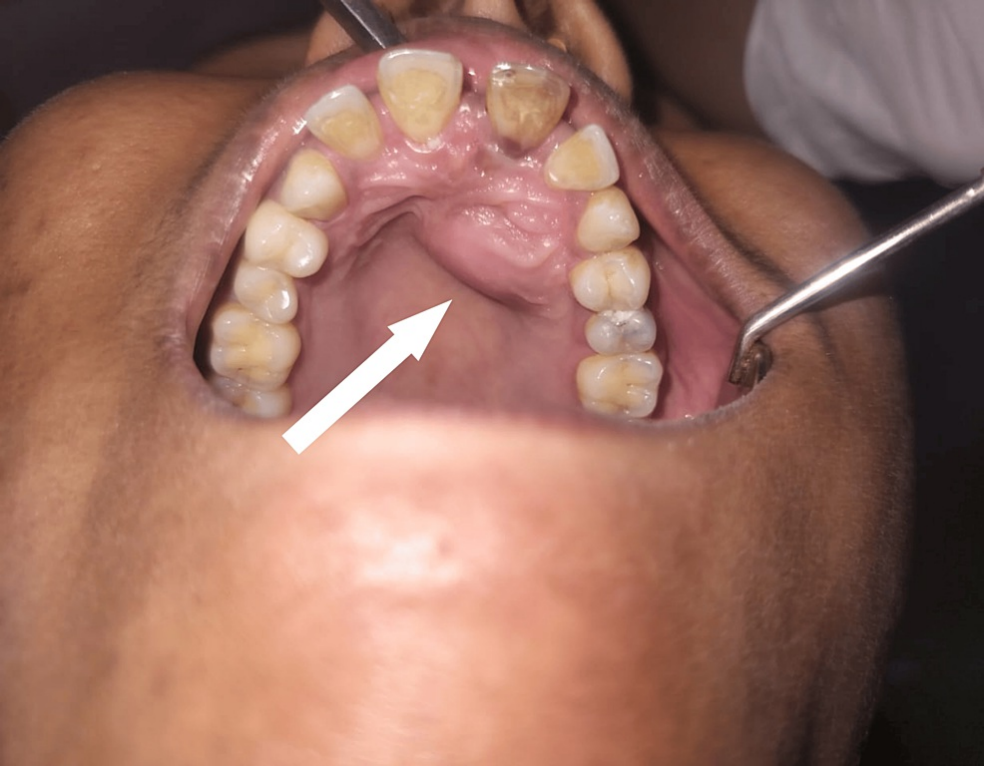
Intraoral examination of the patient showing palatal swelling on the upper front region of the jaw Informed consent from the patient was obtained before taking the photograph of the lesion.

A 26-year-old female presented to the oral medicine department in a tertiary care center in Sawangi, Wardha, India, with a chief complaint of pain in the upper left and front regions of the jaw for two months. The patient had no medical history and was not taking any medication. Dental history was insignificant. On intraoral examination, an incomplete fracture (crack or crazing) of the enamel without loss of tooth structure was observed. Discoloration of the tooth was noted with tooth number 21. Palatal swelling was evident, as shown in Figure 1.

**Figure 2:**
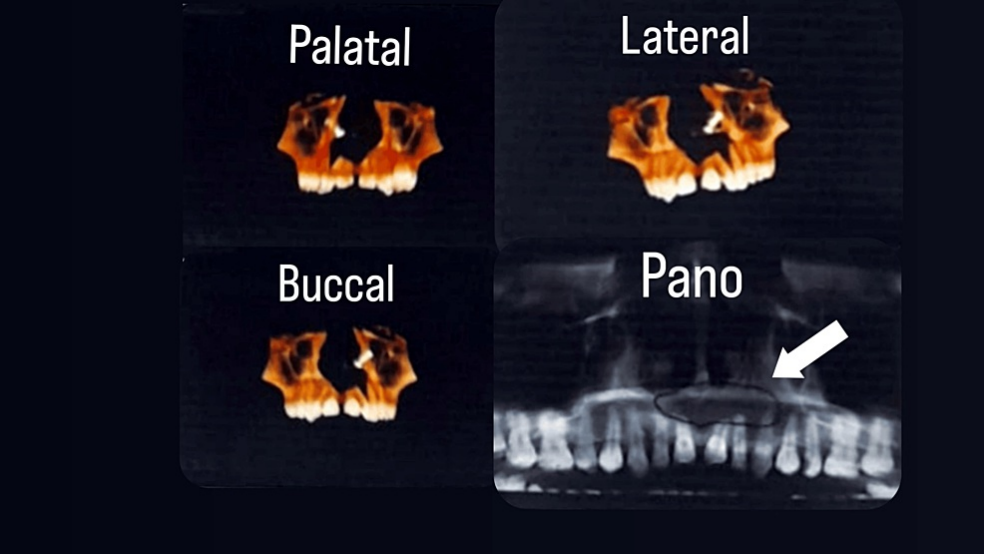
Three-dimensional reconstruction of the lesion

Radiographic findings revealed a unilocular, well-defined expansile hypodense lesion with mild corticated borders present in the maxillary region crossing the midline. It extended from the region of tooth number 11 to the mesial side of tooth number 25. The lesion involved the labial cortical plate, the mid-alveolar cortex region, and the palatal cortical plate, affecting the periapex and apical regions of tooth 11 in the first quadrant, as well as teeth 21 to 24 in the second quadrant, as illustrated in Figure 2. The dimensions of the lesion were approximately 23.11 mm (mesiodistal) × 14.43 mm (buccopalatally) × 21.77 mm (height).

**Figure 3:**
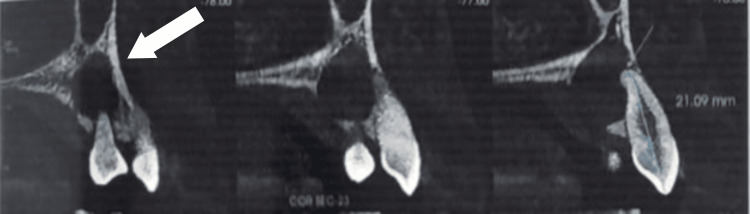
Sagittal view of the lesion in 11 and 12 regions of the tooth

On radiographic impression, thinning of the cortical plates and a breach in the labial cortical plates were noted from tooth 11 in the first quadrant to tooth 24 in the second quadrant. The lesion extended to the mid-alveolar cortex region, involving the palatal cortical plates, with thinning and discontinuity evident. The internal structure appeared completely hypodense, with a loss of trabecular pattern internally. Loss of periodontal ligament was observed from tooth 11 in the first quadrant to tooth 24 in the second quadrant. Loss of the sclerotic border was evident around the nasopalatine canal (NPC), with thinning and discontinuity in the nasal floor. These findings suggested osteolytic lesions involving teeth 11 in the first quadrant and 24 in the second quadrant. The thinning of the labial and palatal cortex was evident in relation to the lesion. The NPC was involved, with a loss of sclerotic border in the lateral aspect and thinning in the nasal floor, as evaluated with tooth 21 (length: 21.09 mm). A radiolucency surrounded by sound radiopaque bone extended from the apex of 21 and 22 regions toward the floor of the nasal cavity, as shown in Figure 3.

A fine needle aspiration was performed, revealing a discharge containing pus and blood. A presumptive diagnosis of an infected radicular cyst was made concerning tooth 11 in the first quadrant and tooth 24 in the second quadrant. Based on the clinical examination, radiographic findings, and aspiration, a differential diagnosis including radicular cyst, traumatic cyst, and periapical abscess was considered for the maxillary region from tooth 11 in the first quadrant to tooth 24 in the second quadrant. Subsequently, a biopsy was sent to the pathology lab to confirm the final diagnosis.

Upon receiving the biopsy report, the diagnosis of a radicular cyst was confirmed. The patient was informed about the etiology of the swelling, and both the patient and her parents were educated about the various treatment modalities available to eliminate the radicular cyst. These treatment options include endodontic therapy and surgical approaches such as marsupialization or enucleation.

Treatment

For root canal treatment and surgical enucleation planned for tooth numbers 11, 21, 22, 23, and 24, the procedure commenced with root canal treatment performed using a round bur (BR-45, Mani, Inc., Utsunomiya, Japan) and a safe end bur (EX 24, Mani, Inc.). The working length was determined using an electronic apex locator. The canals were irrigated with 3% sodium hypochlorite (Vishal Dentocare Pvt Ltd., Ahmedabad, India), followed by 0.9% normal saline to remove any residual pulp tissue and debris. Subsequently, the cavity was disinfected with 2% chlorhexidine to mitigate cytokine reaction induction and halt inflammation spread within the canal, thus reducing inflammation in the periapical space. An intracanal medicament dressing of calcium hydroxide (Prime RC Cal, India) was then placed.

Following root canal treatment, surgical enucleation of the cyst was performed through apicoectomy and retrograde filling of the affected teeth. Local anesthesia was administered, and a full-thickness mucoperiosteal flap was reflected. The surgical site was irrigated with normal saline, and a crevicular incision was made. The cortical bone was widened to ensure thorough curettage. The cyst was carefully curetted, and granulation tissue was excised, followed by irrigation with betadine and normal saline. Subsequently, flap closure was achieved using 3-0 silk sutures, and hemostasis was ensured.

Postoperatively, the patient was provided with instructions, along with a one-week prescription of antibiotics and an analgesic (amoxicillin 825 mg and clavulanic acid 125 mg). The patient was scheduled for postoperative evaluations at one week, six months, and one year following the procedure.

## Discussion

Since epithelial rests are ubiquitous following odontogenesis, odontogenic cysts are common benign lesions of the mandible bones [12]. Out of all odontogenic cysts, the radicular cyst seems to be the most prevalent, occurring in between 50% and 60% of cases. Three stages are generally recognized to comprise the pathogenesis of radicular cysts: initiation, cyst formation, and enlargement [13]. The periodontal ligament’s Malassez epithelial cells rest proliferate through inflammation as a result of bacterial antigens derived from dead pulp and necrotic debris [14].

Studies on cyst fluids and cultured cyst explants from radicular cysts, keratocysts, and follicular cysts revealed that radicular cysts contained higher levels of endotoxins than other types of cysts. Pulpal necrosis, followed by inflammation, seems to be the most common cause of radicular cysts [15]. A less frequent but plausible cause of pulpal necrosis that has been reported in the literature is trauma to the tooth. In our case study, similar findings were found, which resulted in the formation of a radicular cyst. Nonetheless, the patient did report a blunt chin trauma roughly a decade ago. At that time, there were no reports of injuries or bleeding, and no treatment was given. Thus, the pathology appears to have started 10 years ago with a significant trauma. This trauma resulted in the necrosis of the pulp, which led to the formation of the radicular cyst. The anterior maxilla has been the site of most radicular cyst cases reported in the literature. Several theories have been put forth, including the maxillary bone’s sponginess and people’s reluctance to have their anterior teeth extracted because doing so causes cysts to form. Radicular cysts look like small periapical lesions that are usually between 0.1 and 1 cm in size and are associated with one or more carious teeth; however, reports of long-lasting, massive radicular cysts larger than 5 cm have been made [10]. Similarly, compared with our study, a periapical lesion measuring 2.3 cm (mesiodistal) × 1.4 cm (buccopalatally) × 2.1 cm (height) was identified at the most common site (maxillary anterior). This lesion was associated with the tooth from region 11 in the first quadrant extending to the mesial side of tooth 24 in the second quadrant.

## Conclusions

One of the common conditions found in the oral cavity, radicular cysts are asymptomatic when small and are discovered by chance during radiographic examination. This report emphasizes the incidence of symmetrical bilateral radicular cysts and highlights the significance of radiographic examination before tooth extraction. Currently, root canal treatment along with an enucleation surgical procedure is performed to treat periapical cysts. Endodontic therapy, which included thorough irrigation, cleaning, shaping, and obturation of the canal space, was used to successfully manage the current case. The surgical procedure was then performed, which included the administration of local anesthesia followed by enucleation via apicoectomy and retrograde filling of the afflicted teeth, followed by the reflection of a full-thickness mucoperiosteal flap. Then a cervicular incision was made, and curettage was done. The flap closure was done with the help of a 3-0 silk suture. The patient was then prescribed antibiotics and analgesics for one week.
